# Cloning of the canine RNA polymerase I promoter and establishment of reverse genetics for influenza A and B in MDCK cells

**DOI:** 10.1186/1743-422X-4-102

**Published:** 2007-10-23

**Authors:** Zhaoti Wang, Gregory M Duke

**Affiliations:** 1 MedImmune, 297 North Bernardo Avenue, Mountain View, CA 94043, USA

## Abstract

**Background:**

Recent incidents where highly pathogenic influenza A H5N1 viruses have spread from avian species into humans have prompted the development of cell-based production of influenza vaccines as an alternative to or replacement of current egg-based production. Madin-Darby canine kidney (MDCK) cells are the primary cell-substrate candidate for influenza virus production but an efficient system for the direct rescue of influenza virus from cloned influenza cDNAs in MDCK cells did not exist. The objective of this study was to develop a highly efficient method for direct rescue of influenza virus in MDCK cells.

**Results:**

The eight-plasmid DNA transfection system for the rescue of influenza virus from cloned influenza cDNAs was adapted such that virus can be generated directly from MDCK cells. This was accomplished by cloning the canine RNA polymerase I (pol I) promoter from MDCK cells and exchanging it for the human RNA pol I promoter in the eight plasmid rescue system. The adapted system retains bi-directional transcription of the viral cDNA template into both RNA pol I transcribed negative-sense viral RNA and RNA pol II transcribed positive-sense viral mRNA. The utility of this system was demonstrated by rescue in MDCK cells of 6:2 genetic reassortants composed of the six internal gene segments (PB1, PB2, PA, NP, M and NS) from either the cold-adapted (*ca*) influenza A vaccine strain (*ca *A/Ann Arbor/1/60) or the *ca *influenza B vaccine strain (*ca *B/Ann Arbor/1/66) and HA and NA gene segments from wild type influenza A and B strains. Representative 6:2 reassortants were generated for influenza A (H1N1, H3N2, H5N1, H6N1, H7N3 and H9N2) and for both the Victoria and Yamagata lineages of influenza B. The yield of infectious virus in the supernatant of transfected MDCK cells was 10^6 ^to 10^7 ^plaque forming units per ml by 5 to 7 days post-transfection.

**Conclusion:**

This rescue system will enable efficient production of both influenza A and influenza B vaccines exclusively in MDCK cells and therefore provides a tool for influenza pandemic preparedness.

## Background

The type A and B influenza viruses have genomes consisting of eight negative-sense single-stranded viral RNAs (vRNAs), each of which contains a coding region and terminal 5' and 3' noncoding regions. Within the virion, the vRNAs are associated with nucleoprotein (NP) and the three polymerase subunits (PB1, PB2, PA) to form ribonucleoprotein (RNP) complexes. Upon infection of cells, the RNPs are released into the cytoplasm and subsequently enter the nucleus where replication of the vRNA results in the production of both mRNA and complementary RNA (cRNA), the template for synthesis of more vRNA. Errors generated in the viral genome during replication by a low fidelity viral RNA polymerase combined with the segmented arrangement of the influenza genome has resulted in the generation of reassortants in nature with new genetic characteristics. Natural influenza variants have emerged in the past for which humans have little to no immunity and world wide influenza pandemics have ensued.

One aspect of preparation for an influenza pandemic is to create adequate production facilities for vaccine manufacture. Although current production of influenza vaccines for human vaccination is in most cases an egg-base process, many vaccine manufacturers are actively developing cell-based influenza vaccine capabilities. Cell-based influenza vaccine production is potentially less susceptible to biological contamination and more adaptable to large scale production than current egg-based vaccine production. The ability to quickly increase the scale of influenza vaccine production is especially critical in response to an incipient pandemic influenza outbreak, where global vaccination could be an important defence against the development of a full blown pandemic. To this end, MDCK cells are being developed by many of the influenza vaccine manufacturers as a cell-substrate for influenza vaccine production because of the capacity for high virus yields of both A and B strains in this cell line.

In addition to the cell substrate used for influenza virus production, response time to pandemic influenza may be impacted by the need to generate a pandemic vaccine from properly constructed influenza reassortants. These may be reassortants with high growth properties for inactivated vaccine production or reassortants which carry influenza segments that confer attenuation to the resulting pandemic vaccine, either to function as a live vaccine or to reduce risk to production personnel when producing an inactivated vaccine. For any desired reassortant, influenza reverse genetics systems based on rescue of virus from transfected plasmids encoding the viral genome increases the quality, speed, accuracy and reliability of obtaining a desired reassortant as compared to classical reassortment techniques based on dual strain infections followed by selection. Currently, Vero cells are the only cell substrate licensed for plasmid-based rescue of human vaccines. Yet, efficient rescue in Vero cells is hampered by their low productivity for many influenza strains. This prompted us to develop an efficient influenza plasmid-based rescue system in MDCK cells. Although direct influenza A rescue in MDCK cells from a plasmid system based on a T7 RNA polymerase vector has been reported, this system has not been documented to work for type B influenza strains and is less efficient than the eight plasmid bidirectional system which utilizes cellular RNA polymerase I (pol I) and RNA pol II (pol II) to synthesize vRNA and viral mRNA, respectively, from plasmids transfected into susceptible cells [1]. Although efficient rescue occurs in the bidirectional system, the species specificity of the RNA pol I promoter limits its utility to cells from species within the same taxonomic order [2]. The result, which we have confirmed, is that the bidirectional human RNA pol I based plasmid rescue system which functions in primate cells such as Vero, does not support influenza rescue in MDCK cells (data not shown).

For these reasons, we chose to develop a bidirectional influenza reverse genetics system which utilizes a canine RNA pol I promoter derived from MDCK cells as a refinement of the human RNA pol I system developed by Hoffmann and co-workers [3].

## Results and discussion

### Cloning the canine RNA polymerase I promoter from MDCK DNA

In higher eukaryotes, ribosomal RNA (rRNA) genes are transcribed by RNA pol I into a large 45S pre-rRNA which is subsequently processed into mature 18S, 5.8S and 28S rRNAs. Although the mature rRNA sequences are conserved among higher eukaryotes, the 5' non-transcribed region directly upstream of the transcription start site, which contains the RNA pol I promoter and other regulatory sequence elements, has diverged significantly and is conserved only among species within the same taxonomic order [2,4]. Functionally this results in the RNA pol I promoter from a species of one order not being recognized by the RNA pol I and transcription factors from species belonging to other orders. The currently described pol I promoters used in the rescue of recombinant influenza will function only in primate or avian cells [5-7]. In order to rescue influenza using the RNA pol I transcription machinery in canine cells, cloning the canine RNA pol I promoter was necessary.

In developing an approach to cloning the canine RNA pol I promoter, we examined the known features of the RNA pol I promoters and rRNA genes from other mammalian species. In the genomes of human, mouse and rat, the distance from the beginning of the 18S rRNA sequences to the RNA pol I transcription initiation site is 3676, 4026 and 4264 bp, respectively. A BLAST analysis of the pre-rRNA sequences upstream of the 18S rRNA of these species revealed no significant similarities among the sequences (data not shown). Functional assays using cloned regions of DNA have demonstrated that the region immediately upstream of a transcription initiation site has RNA pol I promoter activity *in vitro *and the sizes of these cloned promoter elements have been narrowed to 225 bp and 169 bp for human and mouse genomes, respectively [5,8]. Based on these data, we hypothesized that a functional RNA pol I promoter may be present within a region from 3000 to 5000 bp upstream of the beginning of the canine 18S rRNA gene. Thus, our strategy for isolating the canine RNA pol I promoter was to clone a MDCK genomic DNA fragment that contained sequences which extended at least 5 kb upstream from the start of the 18S rRNA gene sequence. The resulting MDCK clone then would be tested for RNA pol I promoter activity *in vitro*.

**Figure 1:**
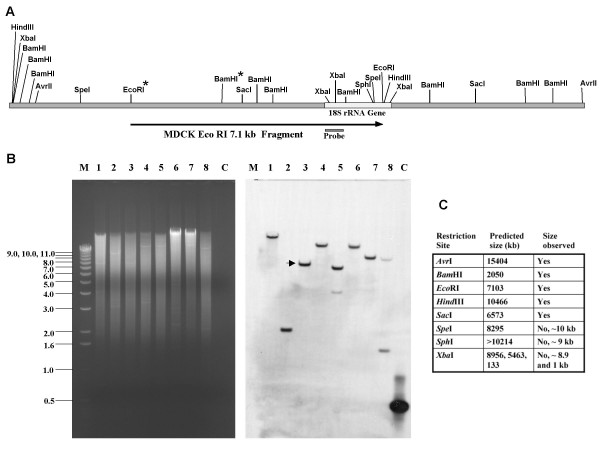
**Restriction enzyme analysis of *canis familiaris *and MDCK DNAs**. (A) Restriction map of the *canis familiaris *genomic sequence [GenBank:NW_878945] which encompasses the 18S r RNA gene. The arrow indicates the position of the MDCK 7.1 kb EcoR I fragment which hybridized to the 18S rRNA gene probe. (B) Southern hybridization of MDCK DNA. Left panel: Single restriction enzyme digestions of MDCK DNA were subjected to electrophoresis on a 0.7% agarose gel and detected by ethidium bromide staining. M: 1 kb ladder (Invitrogen); Lanes 1–8: Avr II, BamH I, EcoR I, Hind III, Sac I, Spe I, Sph I, Xba I; C: 18S rRNA gene probe. Right panel: Southern blot of gel in left panel after hybridization to a psoralen-biotin labeled 18S rRNA gene probe (0.5 kb) and detection by chemilumiscense. The 7.1 kb EcoR I fragment (arrow) was cloned and analyzed for RNA pol I promoter activity. (C) Comparison of the size of selected restriction fragments predicted by the *canis familiaris *genomic sequence to hybridize to the 18S rRNA gene probe and those restriction fragments from MDCK DNA which were observed to hybridize.

The sequence of the region upstream of the canine 18S rRNA initiation site was identified by querying the *canis familiaris *genome with the published canine 18S rRNA sequence [GenBank:AY262732, GenBank:NW_878945]. As expected, analysis of the 5 kb region upstream of the canine 18S rRNA gene showed no significant similarity to human or mouse sequences, nevertheless we suspected that this region contained the canine RNA pol I promoter and transcription initiation site. The predicted restriction sites in the sequence of the *canis familiaris *genome [GenBank:NW_878945], which was derived from a boxer, were used to guide the digestion of MDCK (cocker spaniel) genomic DNA in order to determine the extent of restriction site conservation and to identify restriction fragments expected to contain the RNA pol I promoter (Fig. 1A). These restriction fragments were then probed with18S rRNA sequences in Southern hybridizations (Fig. 1B). Although some of the restriction fragments are conserved in both MDCK and *canis familiaris *DNA (*Avr*II, *Bam*HI, *Eco*RI, *Hind*III and *Sac*I), the Southern results indicated that there was some divergence between these sequences as evinced by the *Spe*I, *Sph*I and *Xba*I digestions, since the predominate fragments hybridizing to the 18S rRNA probe in these digests were not predicted by the *canis familiaris *sequence (Fig. 1C).

**Figure 2:**
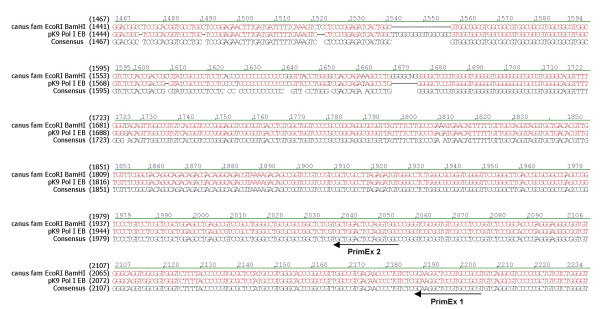
**Alignment of *canis familiaris *sequence and sequences of the MDCK *Eco*RI-*Bam*HI fragment in pK9Pol I EB**. The positions of the primers (PrimEx 1 and PrimEx 2) used in primer extension reactions on MDCK whole cell RNA are indicated by arrows.

Based on the restriction map constructed from the sites conserved between MDCK DNA and the *canis familiaris *genome, the 7.1 kb *Eco*RI fragment which hybridized to the 18S rRNA probe was chosen as a potential RNA pol I promoter candidate since it should be large enough to encompass the pol I promoter based on the relationship between the human pol I promoter, the transcription initiation site and start of the 18S rRNA gene sequence. MDCK DNA was digested with *Eco*RI, subjected to agarose gel electrophoresis and DNA approximately 7 kb in size recovered from the gel. This DNA was ligated into *Eco*RI digested pGEM7 followed by transformation of TOP 10 *E. coli*. DNA preparations from the resulting ampicillin resistant colonies were used as templates in PCR reactions containing forward and reverse primers to the canine 18S rRNA gene. One PCR positive clone (pK9PolI) was identified which upon subsequent sequence analysis confirmed that it contained 18S rRNA sequences and extended approximately 5.5 kb upstream of the 18S rRNA sequences. A 3.5 kbp *Eco*RI-*Bam*HI fragment was subcloned to generate pK9Pol I EB and sequenced. The resulting MDCK sequence was aligned to the *canis familiaris *genomic sequences and they were determined to have 96% identity (Fig. 2).

In order to evaluate the presence of pol I promoter activity in cloned DNA fragments, an MDCK-based assay for replication of an artificial influenza vRNA containing a reporter gene was developed based on an analogous assay used to evaluate the human RNA pol I promoter [9]. The DNA sequences to be tested for RNA pol I activity are cloned upstream of a negative sense reporter gene which has 5' and 3' terminal noncoding sequences derived from influenza vRNA. These noncoding regions of the vRNA in turn enable the antisense reporter transcript to be recognized by influenza replication proteins expressed by co-transfected plasmids and converted into a positive sense transcript which is subsequently translated into a reporter protein, such as enhanced green fluorescence protein (EGFP) or chloramphenicol acetyltransferase (CAT). Additionally, due to the nature of the influenza replication machinery, the transcription initiation and termination sites of this vRNA reporter are critical for functionality, addition of even one extra nucleotide at the 5' end of the negative sense vRNA abrogates the function of this molecule. Therefore, if no pol I promoter element is present in the cloned DNA fragment or if the transcription initiation site is not accurate, no vRNA will result and no reporter signal will be measured.

**Figure 3:**
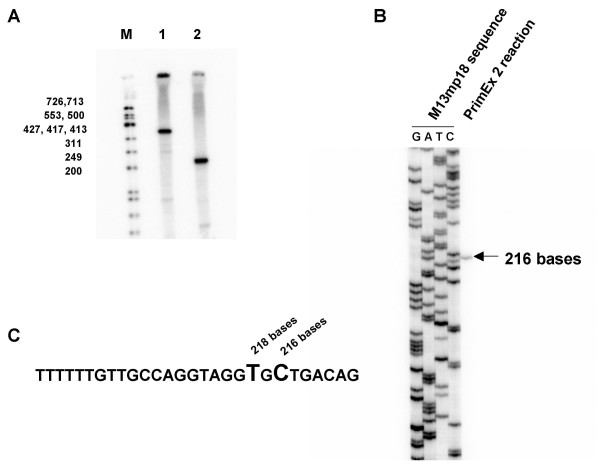
**Determination of the MDCK RNA pol I promoter transcription initiation site by primer extension analysis**. (A) Primer extension reactions on MDCK whole cell RNA using the ^32^P labeled primers PrimEx1 and PrimEx2. Primer extension products and ^32^P ΦX174 size marker DNAs (lane M) were subjected to electrophoresis on a 6% polyacrylamide, 7 M urea gel followed by detection of the radioisotope in the gel with a BioRad Molecular Imager Fx. The maximum length of the observed products were approximately 370 bases and 220 bases, respectively, for the reactions using PrimEx1 (lane 1) and PrimEx2 (lane 2). (B) The products from the PrimEx2 reaction were subjected to electrophoresis adjacent to a M13 sequencing ladder on a 6% polyacrylamide, 7 M urea sequencing gel in order to more accurately determine the maximum length of the products synthesized. (C) The MDCK DNA sequences adjacent to the positions where the largest PrimEx2 products terminated.

In order to determine the position of the rRNA transcription initiation site, ^32^P labeled primers predicted to be <500 bases from the transcription initiation site were used to prime cDNA synthesis on MDCK whole cell RNA. The sizes of the cDNA products from two different primers (PrimEx1 and PrimEx2, Fig. 2) were determined to be approximately 370 and 220 bases, respectively (Fig. 3A). The size of the smaller cDNA product was more accurately determined to be 216 bases by electrophoresis of the cDNA product adjacent to sequencing reactions of M13mp18 DNA, which served as a size ladder (Fig. 3B). In other primer extension reactions, an additional 218 base product also was observed (data not shown).

**Figure 4:**
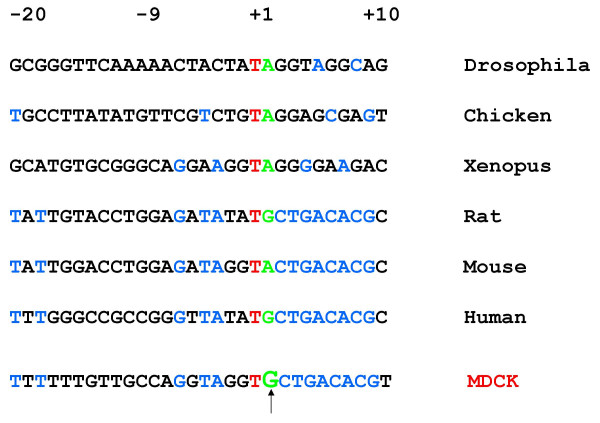
**Comparison of sequences flanking RNA pol I promoter transcription initiation sites**. Sequences adjacent to known RNA pol I promoter transcription initiation sites (nt -20 to + 10) from the indicated species were aligned with MDCK sequences flanking the transcription initiation site mapped by primer extension. The first base of the predominate RNA transcript of the indicated species is labeled +1. Conserved residues are indicated blue, red and green lettering. For all the indicated species, the -1 position is a T (red) and the +1 position a purine residue (green). The sequences from +2 to +9 (blue) are conserved in the indicated mammalian species. Based on the aligned sequences, the G residue (arrow) in the MDCK sequences was predicted to be the RNA pol I promoter transcription initiation site.

**Figure 5:**
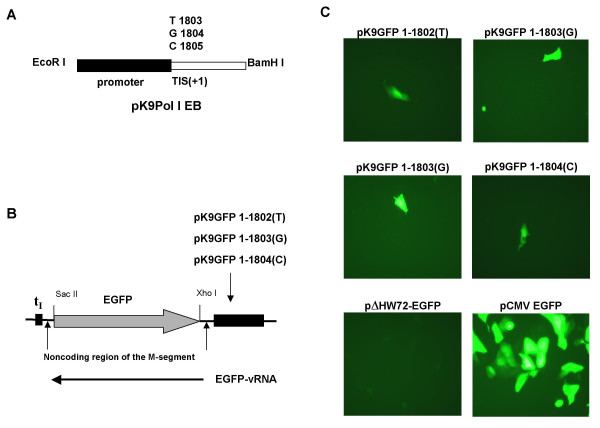
**Replication of artificial vRNA-EGFP reporter transcripts in MDCK cells**. (A) Schematic representation of the MDCK EcoR I- BamH I fragment in pK9Pol I EB. The G residue at position 1804 in the insert was predicted from the alignment in Fig. 4 to be the transcription initiation site (TIS). Products from primer extension reactions designed to map the TIS terminated at T (1803) and C (1805). (B) The EGFP reporter plasmids pK9GFP 1–1802(T), pK9GFP 1–1803(G) and pK9GFP 1–1804(C) were constructed by replacing the human pol I promoter sequences in pHW72-EGFP [9] with bases 1–1802, 1–1803, and 1–1804, respectively, from the MDCK *EcoR *I-*Bam*H I insert in pK9Pol I EB. In each reporter construct, EGFP coding sequences are flanked by the noncoding region from an influenza M segment and this transcription unit is between a murine RNA pol I terminator (**tI**) and the indicated sequences from the MDCK *EcoR *I-*Bam*H I insert. (C) Replication of artificial vRNA-EGFP reporter transcripts in MDCK cells. DNA mixes composed of expression plasmids for PB1, PB2, PA and NP proteins plus a single EGFP reporter plasmid or pΔHW72-EGFP were combined with MDCK cells and subjected to electroporation. At 48 hrs after electroporation, GFP expression was detected by fluorescence microscopy. The plasmid pΔHW72-EGFP is a derivative of pHW72-EGFP in which the human pol I promoter has been deleted. The MDCK cells in the panel labeled pCMV EGFP were subjected to electroporation with pCMV EGFP plasmid alone and served a positive control.

The transcription initiation site was predicted to be either C or T in the sequence shown in Fig. 3C by counting 216 or 218 bases upstream of PrimerEx2 in the MDCK sequence. Yet, when the sequences encompassing this region were aligned to the sequences adjacent to known RNA pol I transcription initiation site sequences of other species, conserved residues in the alignment suggested that the G residue shown in Fig. 4 (arrow) would be transcription initiation site. To address the inconsistency in the primer extension data and the alignments, the MDCK sequences from pK9Pol I EB upstream of the T, G or C residues were individually subcloned into artificial vRNA-EGFP reporter plasmids (Fig. 5A,B). The resulting test reporter constructs are composed of an EGFP gene flanked first by the noncoding regions from an influenza M-segment and then by a murine RNA pol I terminator sequence and the MDCK test sequences. Examination, by fluorescence microscopy, of MDCK cells co-electroporated with expression plasmids for the four influenza replication proteins PB1, PB2, PA and NP plus a single MDCK RNA pol I EGFP reporter construct indicated that use of the MDCK sequences upstream of the G residue in the reporter construct resulted in a RNA pol I synthesized negative sense EGFP transcript which could be replicated to higher levels by the influenza replication proteins, as judged by higher EGFP fluorescent intensity (Fig. 5C) and greater number of fluorescent cells (data not shown). In assays which utilized reporter constructs with the MDCK sequences upstream of the T or C residues, the influenza replication proteins still recognized and replicated the RNA pol I synthesized EGFP transcript, although to lower levels than that observed with the MDCK sequences upstream of the G residue (Fig. 5C). Since authentic influenza sequences without any extra nucleotides are required at the termini of a RNA transcript for it to be replicated efficiently by the influenza replication complex, the results taken as a whole indicate that MDCK RNA pol I predominantly initiates transcription at the G residue in Fig. 4 (arrow) but to a lesser extent utilizes the adjacent T and C residues.

**Figure 6:**
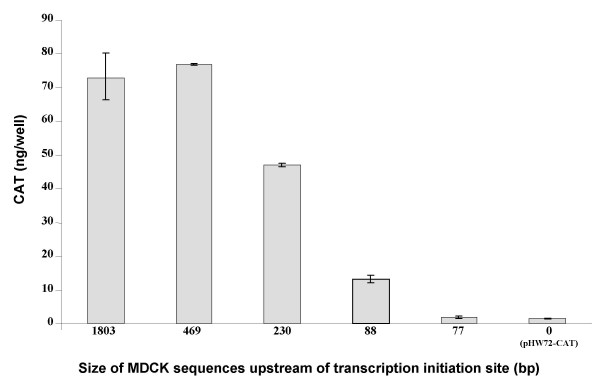
**Evaluation of MDCK sequences required for RNA pol I promoter activity**. As indicated, various lengths of MDCK sequences upstream of the RNA pol I promoter transcription initiation site were cloned into an artificial vRNA-CAT reporter construct. These constructs were individually combined with expression plasmids for PB1, PB2, PA and NP proteins and transfected into MDCK cells. At 44 hrs after transfection, cell lysates were analyzed for CAT expression by a colorimetric ELISA assay. In the plasmid pHW72-CAT, the human RNA pol I promoter directs transcription of a negative sense CAT gene.

The EGFP reporter assay established that a functional MDCK RNA pol I promoter was contained in the 1803 bp sequence upstream of the initiation site. But this sequence is much larger than that required in human and mouse for RNA pol I activity, where the 225 bp (human) or 169 bp (mouse) sequence immediately upstream of the transcription initiation site has pol I promoter activity when transferred into expression constructs [5,8]. To determine if a similar situation held for the MDCK RNA pol I promoter, MDCK DNA fragments which contained sequences that extended various distances upstream of the transcription initiation site were cloned into an artificial vRNA contruct where the EGFP gene had been replaced by a CAT gene. A CAT based system was chosen because CAT expression was found to be capable of detecting differences in RNA pol I activity more accurately than EGFP fluorescence (data not shown). As shown in Fig. 6, the highest level of CAT expression was observed with the construct containing the 469 bp sequence upstream of the transcription initiation site. Reduction to 230 bp and 88 bp upstream of the transcription site lowered CAT expression approximately 35% and 85%, respectively. Finally, the construct with 77 bp upstream of the transcription start site had basal levels of CAT expression similar to the human RNA pol I artificial vRNA construct, pHW72-CAT, which does not contain any MDCK sequences.

**Figure 7:**
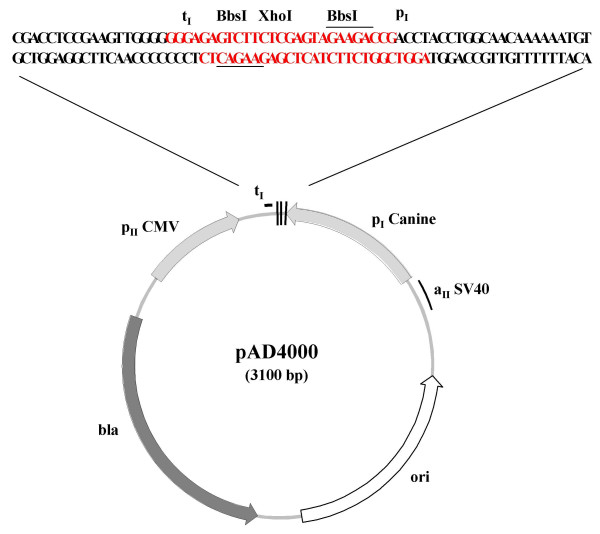
**Map of pAD4000**. The MDCK RNA pol I/CMV pol II bidirectional vector, pAD4000, was derived from pAD3000 [3], the human pol I/CMV pol II bidirectional vector, by replacement of the human pol I promoter sequence in pAD3000 with the MDCK 469 bp sequence upstream of the transcription initiation site. In addition, the two *Bsm*BI restriction sites in pAD3000, which are used for cloning sequences between the two promoters, were changed to *Bbs*I sites because the former restriction site occurs in the MDCK pol I containing fragment.

Based on these results, a MDCK pol I promoter/CMV pol II promoter bidirectional transcription vector was derived from the analogous human RNA pol I promoter/CMV pol II promoter construct, pAD3000, by substitution of the MDCK 469 bp sequence upstream of the transcription site for the human RNA pol I promoter sequence in pAD3000 [3,10]. The resulting construct was designated pAD4000 (Fig. 7). In addition, the two *Bsm*BI restriction sites in pAD3000 which are used for cloning sequences between the two promoters were changed to *Bbs*I sites because the former restriction site occurs in the MDCK RNA pol I promoter containing fragment. Like pAD3000, in pAD4000 there is bi-directional transcription of influenza genomic segments inserted between the two *Bbs*I sites, with the pol I promoter driving expression of influenza vRNA sequences and the CMV pol II promoter directing synthesis of viral mRNA.

### Generation of FluMist^® ^strains from eight plasmids

FluMist is a licensed live attenuated influenza vaccine which currently contains two A virus strains (H1N1 and H3N2) and one influenza B strain. Each vaccine strain component of FluMist is a 6:2 reassortant, composed of the six internal gene segments (PB1, PB2, PA, NP, M, and NS) from an attenuated master donor virus (MDV) strain and the HA and NA gene segments from a wild type (*wt*) strain. The FluMist MDV strains are *ca *A/Ann Arbor/6/60 and *ca *B/Ann Arbor/1/66, originally developed by serial passage at successively reduced temperatures in primary chick kidney cells [11,12].

To demonstrate MDCK RNA pol I plasmid-based rescue, the eight genomic segments of cold adapted (*ca*) A/Ann Arbor/6/1960 (MDV-A) and *ca *B/Ann Arbor/1/1966 (MDV-B) were cloned into pAD4000. Mixtures of eight plasmids (3 μg each) which encoded either the genome of MDV-A or MDV-B were electroporated into MDCK cells. Samples of the media supernatants from the electroporated cells were collected on day 2 – 7 post- electroporation and used in plaque assays to determine virus titer. As shown in Table 1, maximum amounts of virus accumulated in the media on day 4 (1.3 × 10^8 ^pfu/ml MDV-A and 2.3 × 10^7 ^pfu/ml MDV-B).

**Table 1:** Kinetics of MDVA and MDVB generation after transfection^a^

Day post-transfection	Virus titer (pfu/ml)		
	
	MDV-A	MDV-B	MDV-B (mutant)^b^
2	2.8 × 10^4^	1.5 × 10^3^	2.2 × 10^2^
3	2.8 × 10^7^	6.6 × 10^5^	9.8 × 10^4^
4	1.3 × 10^8^	2.3 × 10^7^	5. 2 × 10^6^
5	3.8 × 10^7^	1.9 × 10^7^	1.8 × 10^7^
6	1.2 × 10^7^	3.6 × 10^6^	3. 2 × 10^6^
7	1.2 × 10^7^	2.6 × 10^6^	3. 0 × 10^6^

a Eight plasmids (3ug each) encoding the eight segments of MDV-A or MDV-B were combined with MDCK cells and subjected to electroporation. Virus titer (pfu/ml) of the supernatant was determined at the indicated days after electroporation by plaque assay on MDCK cells.

b The MDV-B mutant was generated by substitution of MDV-B NS and PB1 plasmids containing silent mutations in their coding regions (NS 461A/G, PB1 561T/C, 924A/G) for their MDV-B counterparts in the eight plasmid MDV-B mix.

As a control to demonstrate the rescued virus was derived from the genomic sequences carried in the plasmids, MDV-B NS and PB1 genes containing the silent coding mutations NS (416 A/G) and PB1 (561 T/C, 924 A/G) were also inserted into pAD4000 and substituted for their MDV-B counterparts in an eight plasmid MDV-B mix. Electroporation of MDCK cells with this mix resulted in an accumulation of supernatant virus with kinetics and titer similar to that of the nonmutated MDV-B rescue (Table 1). Subsequent sequence analysis of supernatant virus from the MDV-B NS/PB1 mutant electroporation confirmed the presence of the mutations in the rescued virus and demonstrated that this virus was plasmid derived.

To demonstrate that the MDCK RNA pol I based rescue system was applicable to a wide variety of seasonal FluMist vaccine strains, the HA and NA segments from type A subtypes H1N1and H3N2 plus additional B isolates representing both the Victoria and Yamagata lineages were cloned into pAD4000 and used to rescue 6:2 reassortants in MDCK cells As shown in Table 2, virus titers of approximately 10^6 ^to 10^7 ^were reached by days 5 to 7 post-transfection, which is comparable to results obtained with plasmid-based rescue using the human RNA pol I promoter and a co-culture of primate and MDCK cells [3]. To demonstrate the applicability of this system to influenza isolates which may be precursors of pandemic strains, 6:2 reassortants were rescued in which the HA and NA were derived from highly pathogenic *wt *viruses first isolated from human cases of H5N1 infection in 1997, 2003, and 2004 (Table 2) [13]. For the three *ca *H5N1 reassortants, the cleavage site of the HA gene had been modified by removal of the highly cleavable multibasic amino acid residues, which are a virulence motif in highly pathogenic avian influenza viruses of the H5 and H7 subtypes [14]. In addition, the *ca *H5N1 viruses rescued in this report were previously rescued in Vero cells using human RNA pol I based vectors under enhanced BL-3 containment procedures and subsequently reduced to BL-2 containment status after having been shown to be attenuated in mice, ferrets and chicken. Also, these *ca *H5N1 viruses have been shown to be protective against *wt *H5N1 challenge in mice and ferrets [13]. As such, they are potential candidate vaccines for H5N1 infection and are under further investigation to determine whether they are appropriate for human use.

**Table 2:** Plasimid Rescued influenza A & B Viruses In MDCK cells^a^

Type	Strain	Virus titer (pfu/ml)
A		
H1N1	ca A/New Caledonia/20/1999	7. 0 × 10^6^
	ca A/Solomon Island/3/2006	5.4 × 10^7^
H2N2	MDV-A (ca A/Ann Arbor/6/1960)	1.5 × 10^8^
H3N2	ca A/Wisconsin/67/2005	8.4 × 10^6^
	ca A/California/7/2004	2.6 × 10^6^
	ca A/Panama/2007/1999	9. 0 × 10^6^
H5N1	ca A/Hong Kong/213/2003	1.3 × 10^7^
	ca A/Hong Kong/1997(491 H5/486 N1)	1.7 × 10^7^
	ca A/Vietnam/1203/2004	4.6 × 10^7^
H6N1	ca A/Teal/HK/W312/1997	6.0 × 10^6^
H7N3	ca A/CK/BC-CN/2004	3.9 × 10^6^
H9N2	ca A/chicken/HK/G9/1997	1.4 × 10^7^
B		
	MDV-B (B/Ann Arbor/1/1966)	6.0 × 10^7^
	MDV-B (B/Ann Arbor/1/1966)-Mutant	1.5 × 10^7^
Victoria	ca B/Hong Kong/330/2001	1.7 × 10^7^
	ca B/Malaysia/2506/2004	1.6 × 10^7^
Yamagata	ca B/Jiangsu/10/2003	5.3 × 10^7^
	ca B/Florida/07/2004	2.6 × 10^7^

a Supernatants of transfected MDCK cells were collected on days 5, 6, and 7 after transfection. Virus titer was determined by plaque assay for the supernatant collected on the day post-transfection when the MDCK cells exhibited 50 to 100% CPE.

To further test the MDCK based system, 6:2 reassortants were rescued in which the HA and NA were derived from isolates of the H6N1, H7N3 and H9N2 subtypes (Table 2). The *wt *virus which was the source of the HA and NA for the H6N1 reassortant was isolated from teal and contains seven segments (NA, PB1, PB2, PA, NP, NS and M) which are very similar to their counterparts in the human H5N1 virus A/Hong Kong/156/97 and it may represent a derivative or precursor of the H5N1 viruses [15]. The H7N3 *wt *virus (A/CK/BC-CN/2004) exhibits low pathogenicity in avian species although it was isolated during an outbreak of a related highly pathogenic H7N3 strain which contained a multibasic amino acid insertion near the HA0 cleavage site [16]. Finally, the H9N2 virus (A/chicken/HK/G9/1997) is representative of one of the three H9N2 subgroups which were isolated from poultry in Hong Kong during the 1997 H5N1 outbreak in humans [17].

## Conclusion

Using the MDCK RNA pol I plasmid-based system, we have demonstrated the rescue of a wide variety of influenza A subtypes as well both lineages of influenza B. These results indicate that influenza reverse genetic performed exclusively in MDCK cells can efficiently result in the rescue of seasonal FluMist vaccine strains as well as prototype attenuated vaccines for *wt *strains which may harbor the potential for becoming pandemic. The application of the rescue system utilizing the MDCK RNA pol I promoter reported here is focused on the rescue of FluMist influenza vaccine strains in MDCK cells, although our expectation is that it should be applicable to other influenza strains too. As such, this refinement of the plasmid-based rescue system for influenza virus may be a useful tool in the development of vaccines as a response to an imminent pandemic.

## Methods

### Nucleic acid extraction and Southern hybridization

Total DNA and RNA was recovered from MDCK cells (passage 64, ATCC) using MasterPure™ DNA Purification Kit and MasterPure™ RNA Purification Kit, respectively, according to the manufacturer's instructions (Epicentre Biotechnologies). For Southern hybridization experiments, MDCK DNA (20 μg) was digested overnight at 37°C with the indicated restriction enzyme and subjected to electrophoresis on 0.7 % agarose gels. DNA was transferred to Hybond-N+ membranes (Amersham Corp.) and immobilized with a UV Crosslinker 10000 (Hoeffer Scientific Instruments).

Probe DNA was prepared by PCR amplification of sequences from the 5' end of the 18S rRNA gene using MDCK DNA as the template and forward and reverse primers (5'-CTTGTCTCAAAGATTAAGCCATGCATG-3' and 5'-CAGGGCCTCGAAAGAGTCCTGTATTG-3', respectively). The PCR products were labeled using a BrightStar Psoralen-Biotin Nonisotopic Labeling Kit according to the manufacturer's instructions (Ambion) and hybridizations were performed as described previously [18]. Detection of hybridized probe DNA was performed using a BrightStar BioDetect TM Nonisotopic Detection Kit (Ambion).

### Cloning MDCK DNA and plasmid construction

All cloning and PCR reactions were performed according to standard protocols. To clone the 7.1 kb MDCK *Eco*R I fragment which hybridized to 18S rRNA sequences, 100 μg of MDCK DNA was digested with 100 units of *Eco*R I overnight at 37°C and subjected to electrophoresis on a 0.7 % agarose gel along with a 1 kb ladder size marker. Using the marker as a guide, the 7 kb region of the *Eco*R I digested MDCK DNA lane was excised from the gel followed by recovery of the DNA from the gel sample. The recovered DNA was ligated to *Eco*R I digested pGEM 7 vector (Promega) and the ligation mixture was used to transform *E. coli *TOP10 cells (Invitrogen). DNA preparations from the resulting ampicillin resistant colonies were used as templates in PCR reactions containing the same forward and reverse primers to the canine 18S rRNA gene that were used to prepare the probe for the Southern hybridizations. PCR products then were analyzed on agarose gels to identify colonies which produced 500 bp products, the size predicted from the 18S rRNA gene sequence. One such clone, designated pK9PolI, was determined by nucleotide sequencing and restriction enzyme analysis to have a 7.1 kb insert which contained canine 18S rRNA sequences. Plasmid pK9Pol I EB was constructed by subcloning the 3.5 kb *Eco*RI *Bam*HI fragment from the insert contained in pK9Pol I into pGEM 7.

Reporter plasmids pK9GFP 1–1802(T), pK9GFP 1–1803(G), and pK9GFP 1–1804(C) were derived from pHW72-EGFP [9] by replacing the human RNA pol I promoter sequences in pHW72-EGFP with bases 1–1802, 1–1803 and 1–1804, respectively, from the MDCK *Eco*RI *Bam*HI insert in pK9Pol I EB. Reporter plasmid pK9CAT(1803) was derived from pK9GFP 1–1803(G) by replacing the EGFP gene with a CAT gene. The plasmids pK9CAT(469), pK9CAT(230), pK9CAT(88) and pK9CAT(77) were made by deleting, respectively, the MDCK sequences 1–1334, 1–1573, 1–1715 and 1–1726 from the 1803 bp MDCK-derived sequence in pK9CAT(1803), where position 1803 is at -1 with respect to the RNA pol I transcription start site. The plasmid pΔHW72-EGFP is a derivative of pHW72-EGFP in which the human pol I promoter has been deleted.

The plasmid for expression of influenza segments in MDCK cells, pAD4000, was derived from pAD3000 [3] by replacing human RNA pol I promoter sequences in pAD3000 with the MDCK RNA pol I promoter sequences from pK9CAT (469). In addition, the cloning sites between the RNA pol I promoter and the RNA pol I terminator were changed from *Bsm*B I-*Kpn *I-*Bsm*B I (pAD3000) to *Bbs *I-*Xho *I-*Bbs *I (pAD4000).

Influenza segments, previously cloned into pAD3000 and shown to be virus rescue competent [3,10,19] were amplified with Accu Prime Pfx DNA Polymerase (Invitrogen) using forward and reverse primers containing, respectively, segment specific 5' or 3' sequences and a restriction site appropriate for cloning between the *Bbs *I sites of pAD4000. For influenza A strains, the restriction sites were *Bsm*BI (PB1, PA, NP, M, and NS) or *Aar*I (PB2, HA and NA). For influenza B strains, the restriction sites were *Bsm*BI (PB1, PB2, PA, HA, NA, M, and NS) or *Aar*I (NP). The HA and NA segments subcloned from pAD3000 into pAD4000 were originally derived from the following *wt *strains: A/New Caledonia/20/1999 (H1N1), A/Solomon Island/3/2006 (H1N1), A/Wisconsin/67/2005 (H3N2), A/California/7/2004 (H3N2), A/Panama/2007/1999 (H3N2), A/Hong Kong/213/2003 (H5N1), A/Hong Kong/1997(491 H5/486 N1), A/Vietnam/1203/2004 (H5N1), A/Teal/HK/W312/1997 (H6N1), A/BC-CN/04 (H7N3), A/chicken/HK/G9/1997 (H9N2), B/Malaysia/2506/2004, B/Jiangsu/10/2003, B/Hong Kong/330/2001 and B/Florida/07/2004.

### Primer extension reactions and M13 sequencing

Primers for primer extention reactions (PrimerEx1: 5'-CGCGGCACGGAGCCTTGC-3' and PrimerEx2: 5'-GCCACCTGGAGTCCAGCA-3'), M13 (-40) sequencing primer and dephoshorylated, *Hin*f I digested φX174 size marker DNAs were labeled at their 5' ends with [γ-^32^P] ATP using T4 polynucleotide kinase for 30 min at 37°C. After labeling, ^32^P PrimerEx1 and ^32^P PrimerEx2 were used to direct cDNA synthesis from MDCK whole cell RNA utilizing a Primer Extension System-AMV Reverse Transcriptase kit (Promega). Primer extension products and ^32^P labeled φX174 size marker DNAs were subjected to electrophoresis on 6% polyacrylamide, 7 M urea gels followed by detection of the radioisotope in the gel with a BioRad Molecular Imager Fx. PrimerEx2 primer extension products were also subjected to electrophoresis adjacent to a M13 sequencing ladder on a 6% polyacrylamide, 7 M urea sequencing gel followed by imaging of the radioisotope in the gel. Sequencing reactions using ^32^P labeled M13 (-40) sequencing primer and M13mp18 ssDNA template were performed utilizing a Sequenase Version 2.0 DNA sequencing kit (USB).

### Cell culture and transfection

MDCK cells were originally obtained from the American Type Culture Collection and were maintained in MEM supplemented with 10% fetal bovine serum (FBS). One day prior to electroporation, subconfluent MDCK cells were detached from their growth flasks by treatment with trypsin and seeded at 5 × 10^4 ^cells/cm^2^. The following day, cells were detached from their growth flask by treatment with trypsin, pelleted by centrifugation and resuspended in Opti MEM I (Invitrogen). For each electroporation, 5 × 10^6 ^cells were mixed with Opti-MEM I to a final volume of 300 ul in a 0.4 cm electroporation cuvette (BioRAD). DNA (3 μg of each individual plasmid in no more than 25 μl) was added to the cells in the cuvette followed by electroporation at 220 volts, 950 microFarads (BioRad Gene Pulser II). Opti-MEM I (0.7 ml) was added to the cuvette about 2 min after electroporation, gently mixed with the DNA-cell mixture and transferred to a well of a 6 well plate which contains 2 ml Opti-MEM I. After incubation at 33°C for 16–20 hrs, cells were washed with 2 ml of Opti-MEM I to remove unattached cell debris and overlayed with 2 ml of Opti-MEM I containing 1 ug/ml TPCK-trypsin. On days 2–7 post-electroporation, a 1 ml sample of media was collected from the electroporated cells and replaced with 1 ml Opti-MEM I containing 1 ug/ml TPCK-trypsin. Virus in the collected samples was titrated by plaque assay on MDCK cells followed by immunostaining with an influenza virus-specific polyclonal antibody and plaques were visualized using a secondary antibody conjugated with horseradish peroxidase.

Transfections using PromoFectin (PromoKine, Heidelberg, Germany) were performed according to the manufacturer's protocol with slight modification. Briefly, MDCK cells (1.5 × 10^5 ^cells/ml) in MEM supplemented with 10% FBS were seeded at 2 ml per well of a 6 well plate approximately 24 hrs before transfection. DNA (3 ug total) was diluted into 100 ul of Opti-MEM I. A mix composed of 8 ul of PromoFectin in 100 ul of Opti-MEM I was added to the diluted DNA and immediately mixed using a vortex mixer. After 30 min at room temperature, the PromoFectin/DNA mixture was added drop-wise to one well of MDCK cells (50 to 60% confluent) and the mixture was distributed by gently swirling. The plate then was subjected to centrifugation at 280 g for 5 min at room temperature and incubated at 33°C. After 4 to 5 hrs of incubation, the transfection mix was removed and the cells were washed with 2 ml/well Opti-MEM followed by the addition of 1.5 ml/well of Opti-MEM I containing 0.5 ug/ml TPCK-trypsin. Incubation was continued at 33°C with the addition 1 ml of Opti-MEM I containing 1 ug/ml TPCK-trypsin on days 2 and 4 after transfection. Microscopic examination of the transfected cells was performed daily to check for the appearance of CPE (characteristic virus induced cytopathic effect). The media from wells exhibiting 50 to 100% CPE (usually 5 -7 days after transfection) was collected and virus titrated by plaque assay on MDCK cells followed by immunostaining, as described above, to confirm the influenza virus infection.

### CAT assay

Cell lysates were prepared from transfected cells approximately 44 hours post-transfection and assayed for the synthesis of CAT using CAT ELISA kits (Roche Applied Science) according to the manufacturer's protocol.

## Competing interests

The author(s) declare that they have no competing interests.

## Authors' contributions

GMD is the primary author of this manuscript and is responsible for the overall design of the experiments. ZW assisted in experimental design and was responsible for the performance of all experiments except for the primer extension analysis, which was performed by GMD. All authors have read and approved the final manuscript.
